# Long-term in vivo imaging reveals tumor-specific dissemination and captures host tumor interaction in zebrafish xenografts

**DOI:** 10.1038/s41598-020-69956-2

**Published:** 2020-08-06

**Authors:** Nandini Asokan, Stephan Daetwyler, Stefanie N. Bernas, Christopher Schmied, Steffen Vogler, Katrin Lambert, Manja Wobus, Martin Wermke, Gerd Kempermann, Jan Huisken, Michael Brand, Martin Bornhäuser

**Affiliations:** 1 Center for Regenerative Therapies Dresden (CRTD), Technische Universität Dresden, Fetscherstraße 105, 01307 Dresden, Germany; 2 Division of Hematology, Oncology and Stem Cell Transplantation, Medical Clinic I, Department of Medicine I, University Hospital Carl Gustav Carus, Technische Universität Dresden, Fetscherstraße 74, 01307 Dresden, Germany; 3 Max Planck Institute of Molecular Cell Biology and Genetics (MPI-CBG), Dresden, Germany; 4 German Center for Neurodegenerative Diseases (DZNE), Dresden, Germany; 5 National Center for Tumor Diseases (NCT), Dresden, Germany; 6 German Consortium for Translational Cancer Research (DKTK), DKFZ, Heidelberg, Germany; 7 Present Address: Morgridge Institute for Research, Madison, USA

**Keywords:** Breast cancer, Cancer imaging, Cancer models, Acute myeloid leukaemia

## Abstract

Understanding mechanisms mediating tumor metastasis is crucial for diagnostic and therapeutic targeting. Here, we take advantage of a transparent embryonic zebrafish xenograft model (eZXM) to visualize and track metastatic cells in real time using selective plane illumination microscopy (SPIM) for up to 30 h. Injected human leukemic and breast cancer cells exhibited cell-type specific patterns of intravascular distribution with leukemic cells moving faster than breast cancer cells. Tracking of tumor cells from high-resolution images revealed acute differences in intravascular speed and distance covered by cells. While the majority of injected breast cancer cells predominantly adhered to nearby vasculature, about 30% invaded the non-vascularized tissue, reminiscent of their metastatic phenotype. Survival of the injected tumor cells appeared to be partially inhibited and time-lapse imaging showed a possible role for host macrophages of the recipient embryos. Leukemic cell dissemination could be effectively blocked by pharmacological ROCK1 inhibition using Fasudil. These observations, and the ability to image several embryos simultaneously, support the use of eZXM and SPIM imaging as a functional screening platform to identify compounds that suppress cancer cell spread and invasion.

## Introduction

Tumor metastasis is a highly dynamic, complex, and multistage process during which primary tumor cells disseminate from their site of origin, intravasate, and then leave the blood stream to invade distant organs^1,2^. Metastases represent a major determinant of cancer-associated morbidity and death, and an in-depth understanding of the steps involved in this process will lead to improved diagnostic and therapeutic regimens. Even though several in vitro models have been developed to dissect the metastasis cascade, they do not adequately mimic the in vivo complexity of this process. Existing in vivo models and various in vivo imaging tools, such as intra-vital imaging^3–8^, magnetic resonance imaging^9^, two-photon/multiphoton laser scanning microscopy^10,11^, and optical frequency domain imaging^12^, have played a pivotal role in studying certain facets of metastatic spread, allowed short-term tracking of individual cells, provided unique insights into the mechanism of tumor invasion by capturing migration, plasticity of single cells and their microenvironments, and associated changes in gene expression. Although these advances have enabled a better understanding of metastasis, existing models are not amenable to visualization and continuous monitoring of tumor cells in real time. A long-term tracking of individual cells is, however, required to better understand the metastasis process and to determine the homing pattern of cells in specific tumor microenvironments. The previously mentioned imaging techniques have inherent limitations in long-term tracking as some of them rely on surgical dissection to expose the imaging site, leading to animal survival issues due to surgery, side-effects of extended exposure to anesthetics, and tissue dehydration^8^ or do not provide the required imaging speed to continuously monitoring tumor cells in real time. Moreover, their translational relevance is limited as they are mostly based on murine tumors.

To understand tumor progression and dissemination in vivo in animals without survival issues, the zebrafish is increasingly used as model system^13,14^. Characteristics of zebrafish such as its optical clarity during embryogenesis, availability of pigment-deficient fish lines^15^, and amenability to transplantation assays^16^ make zebrafish a versatile animal model for long term imaging studies^17^ and cancer research^18–20^. Importantly, gene expression profiles of human and zebrafish cancers, such as liver cancer, leukemia (T-ALL), and melanoma, show a high degree of similarity, suggesting evolutionary conservation of pathways associated with cancer progression^21,22^. Also, various human tumor cell lines, including melanoma, glioma, hepatoma, lung cancer, pancreatic cancer, ovarian carcinomas, breast cancer, prostate cancer, retinoblastoma, and leukemia, have been xenotransplanted into zebrafish^23^ to study several aspects of tumorigenesis, like tumor cell migration, angiogenesis, extravasation, and micrometastases^24–28^. Most importantly, proof-of-concept studies have suggested that xenogeneic tumor transplant models using zebrafish embryos can be used as a screening platform to identify novel therapeutic compounds and approaches^29–32^. However, long-term, high resolution, time-lapse images of such transplanted tumor cells are lacking, and their behavior in circulation has not been continuously monitored. This information would be very valuable and help analyze the dynamics of tumor cell spread and invasion in real-time.

Therefore, to understand the dissemination and behavior of tumor cells in circulation, we utilized an existing embryonic zebrafish xenograft model (eZXM). Here, we describe the use of high-resolution, non-invasive selective plane illumination microscopy (SPIM) to visualize the characteristics of tumor cells in real time and investigate the metastatic process in vivo. The SPIM images revealed adaptations in tumor cell morphology in accordance with their surrounding environment. Using an in-house developed, semi-automated tracking method, we could identify distinct intravascular migration patterns and provide, for the first time, tumor-specific speed and distance measurements. Further, we show that the transplanted tumor cells undergo extravasation and invade the host embryo. Finally, we also demonstrate the suitability of our model for therapeutic intervention using the anti-leukemic drug, Fasudil, a ROCK1 inhibitor. These aspects, combined with the versatility in imaging techniques, make the zebrafish an ideal platform for direct and continuous in vivo observation of tumor cells to enable a better understanding of tumorigenesis.

## Results

### Dissemination of cancer cells in vasculature

**Figure 1:**
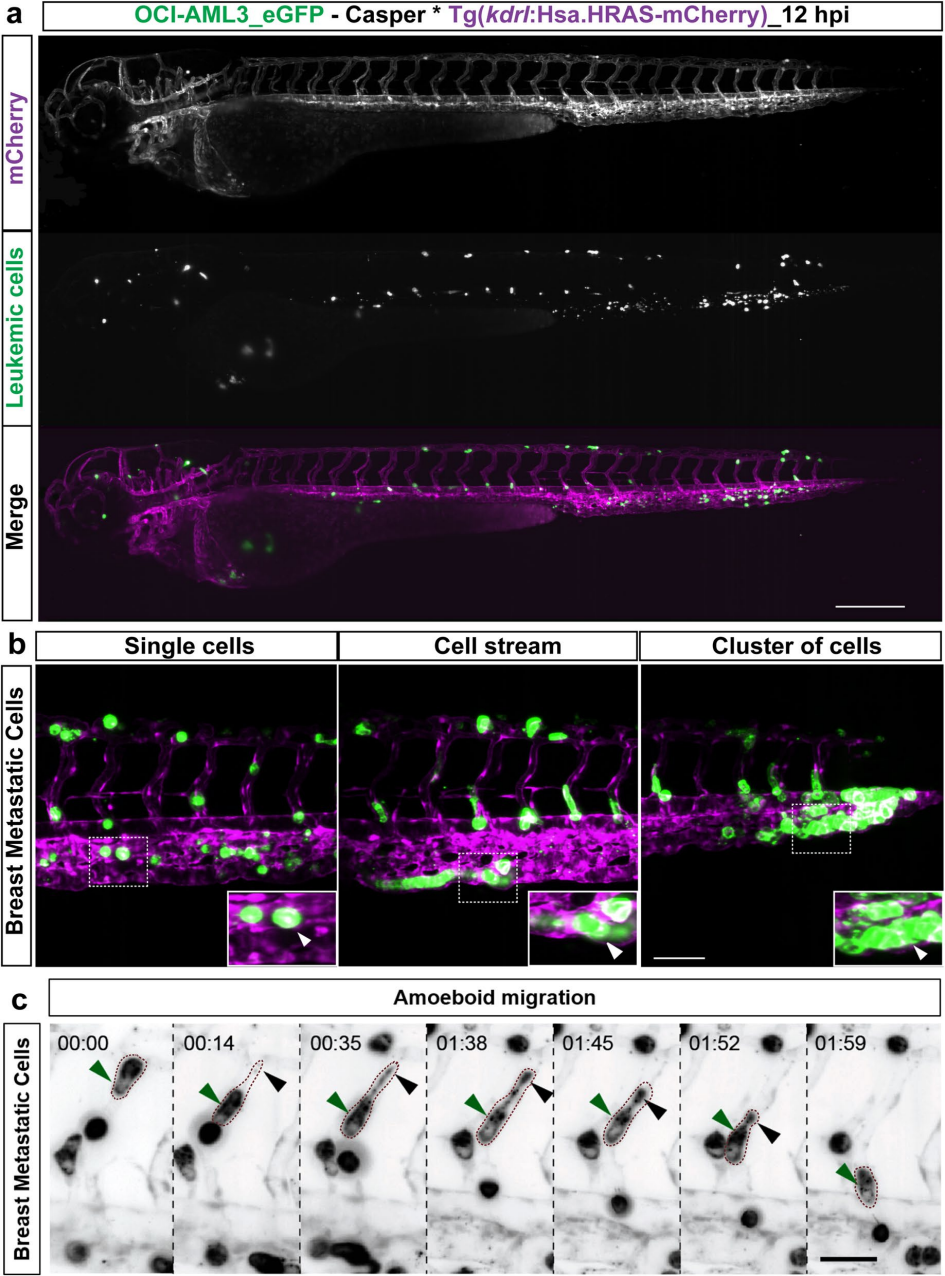
Dissemination and migration modes of tumor cells. (**a**) Snapshot from time-lapse movie of eZXM expressing the vascular marker *Tg*(*kdrl:Hsa.HRAS-mCherry*) in *casper* background (magenta label) injected with eGFP labeled leukemic cells (OCI-AML3_eGFP) (green label). The cells disseminated throughout the embryo. Scale bar: 500 µm. (**b**) High-magnification SPIM revealed diverse migratory modes of breast tumor cells. Representative images of tumor cells migrating either as single cells (left), loosely attached cell streams (center), or cluster of cells (right) indicated by white arrowheads. Insets showed the higher magnification of dotted boxes; Vasculature in magenta, breast tumor cells in green; scale bar 100 µm. (**c**) A breast tumor cell (MDA-MB-231, green label, green arrowheads) migrating through an intersegmental vessel (magenta label) in an amoeboid fashion (as indicated by dashed brown border). The cell formed a large protrusion, with a filopodia-like arm at the trailing end (black arrowheads). Time shown as h:min, scale bar 50 μm.

We utilized early stage embryos (48 hpf) expressing the green fluorescent vascular marker^33^*Tg*(*kdrl:EGFP*)^*s843*^ or the mCherry fluorescent vascular marker *Tg*(*kdrl:Hsa.HRAS-mCherry*)^34^ on a *casper* background for in vivo visualization of engraftment^15^. A multi-sample, multidirectional SPIM^35,36^ was used to monitor and characterize the dissemination profiles in real-time of the triple negative breast cancer cell line MDA-MB231 (representative solid tumor-cell xenograft,injected into *Tg*(*kdrl:EGFP*)^*s843*^ embryos) and a leukemic cell line OCI-AML3_eGFP (representative leukemic xenograft, injected into *Tg*(*kdrl:Hsa.HRAS-mCherry* embryos). Time-lapse images were acquired for up to 30 h for the respective cell types and analyzed. Interestingly, we found that irrespective of the tumor cell type injected, all cells disseminated similarly throughout the embryo from head to tail (DoC; Movie_1 & 2; Fig. 1a). After 5 hpi, we observed that while the solid tumor cells preferred to adhere to nearby regions, leukemic cells tended to migrate continuously. To understand the migration patterns and morphological changes in individual cells, higher magnification time-lapse images of injected embryos were acquired. These images revealed that in vivo, irrespective of cell type, tumor cells migrated as a combination of individual cells, cell streams, or clusters (only breast cancer cells), and that only few cells stayed non-motile and became adherent after homing to one site (Fig. 1b). Tumor cells stayed intravascularly after injection. Higher magnification images also revealed that solid tumor cells (breast cancer) present at the dorsal longitudinal anastomotic vessels (DLAVs) showed an amoeboid type of migration to migrate to the caudal hematopoietic tissue via the intersegmental vessels (ISV). The amoeboid-like migration was characterized by formation of large protrusions with an elongated spindle shaped cell body and filopodia like structures at the trailing end (facing towards DLAVs) (Fig. 1c). However, non-migrating breast cancer cells in ISVs and other migrating cells from parts other than ISV, were found to be round and compact in shape. In contrast to solid tumor cells, leukemic cells mostly kept a spherical shape.

### Leukemic cells display rapid intravascular dissemination

**Figure 2:**
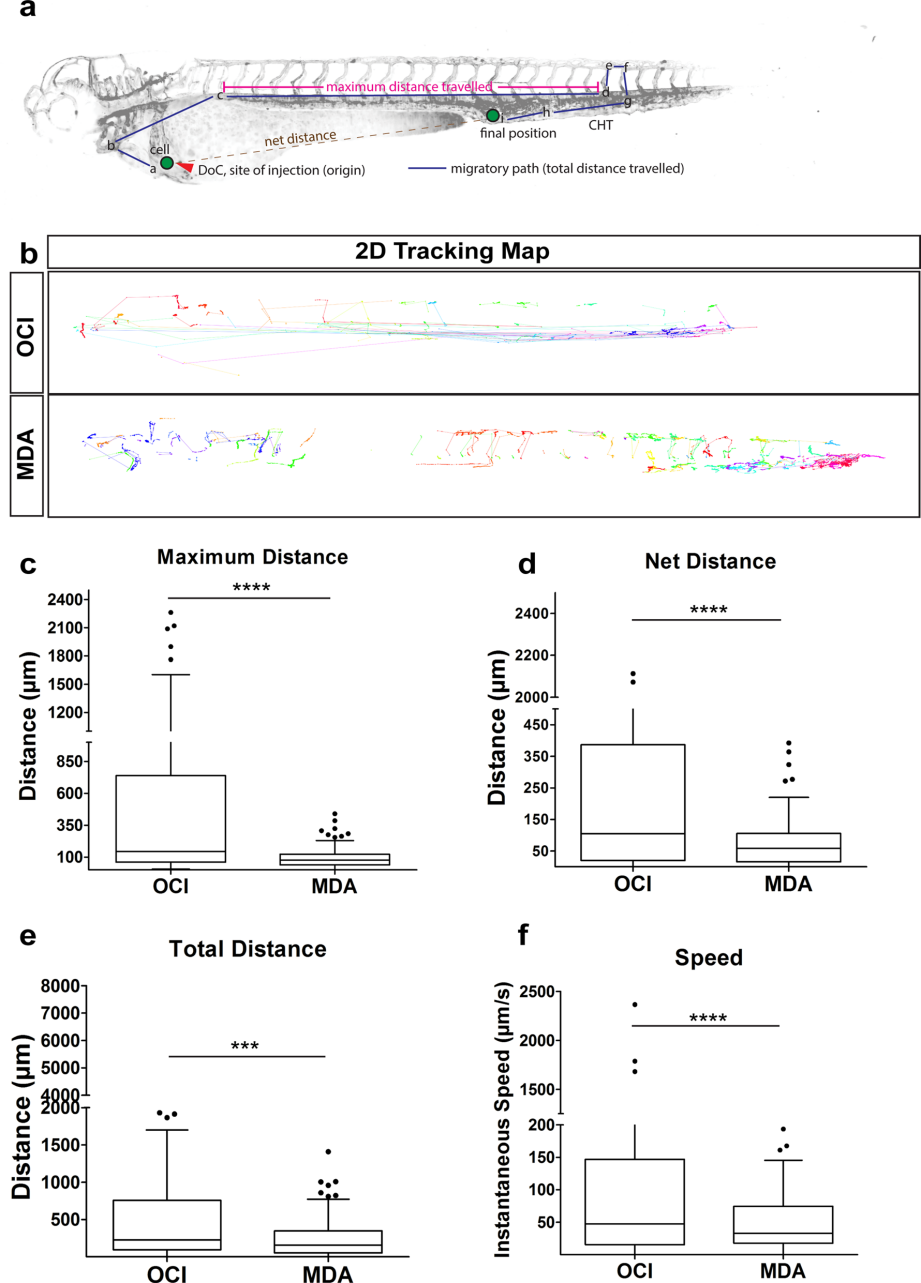
Tumor cell tracking revealed dynamics of xenografted cells. (**a**) Schematic of the quantified dissemination characteristics of a xenografted cell (green). DoC Duct of Cuvier, CHT caudal hematopoietic tissue. (**b**) 2D tracking map of the migratory path taken by every cell in vivo using semi-automated tracking analysis of the SPIM time-lapse movies. The tracking map revealed circulatory paths of leukemic cells (OCI, top), short migratory paths of breast tumor cells (MDA, bottom). Each color represented an individual cell. (**c**–**f**) Representative plot of R-analysis of the cell tracking revealed that (**c**) the maximum distance between any two time points was higher in leukemic cells (OCI) compared to either breast tumor cells (MDA). (**d**) The net distance was significantly higher for leukemic cells (OCI) compared to breast tumor cells (MDA (**e**) Leukemic cells (OCI) covered a significantly greater total distance compared to breast tumor cells (MDA). (**f**) Intravascular speed measurements in both cell types: breast tumor cells (MDA), and leukemic cells (OCI) revealed that OCI showed fastest migration. (**c**–**f**) Plots represent means ± sem.[N = 3 embryos; for each embryo ~ 150 to 200 cells were analyzed] Statistical analyses: (**c**–**f**) Kruskal–Wallis one-way analysis of variance were performed followed by post hoc Dunn’s method for multiple comparisons. Multiple comparisons: (**c**–**f**) OCI vs. MDA.

Direct in vivo recording of SPIM data from the eZXM allowed us to quantitatively analyze parameters of cell dissemination. We applied an in-house developed tracking method for the cells in anterior–posterior and dorsal–ventral direction, combined with manual correction of the tracking results. Dissemination characteristics were described in terms of maximum and total distance travelled, and net distance. Maximum distance travelled was defined as the largest distance between any two given time points in the cell’s migratory path, net distance as the distance separating a cell’s first (origin) and last (final) positions over the entire movie of 30 h, and the total distance travelled as the path taken by the cells from their origin to their final position (Fig. 2a).

We found that migration patterns, distance, and speed of migration exhibited cell-type specific behavior. Specifically, breast cancer cells moved relatively shorter distances compared to leukemic cells. Further, leukemic cells showed faster intravascular dissemination and preferred to stay in circulation (depicted by straight lines in the 2D tracking map). Breast cancer cells, however, tended to adhere to one site, specifically to the caudal hematopoietic tissue (CHT) of the zebrafish embryo (Fig. 2b). Concurring with visual observations from SPIM movies, we found that a leukemic cell covered a significantly longer maximum distance travelled in anterior–posterior and dorsal–ventral direction (459.0 ± 70.05 µm) compared to breast tumor cells (91.44 ± 6.08 µm; *P* < 0.0001) (Fig. 2c). Moreover, leukemic cells displayed a significantly higher net distance (in anterior–posterior and dorsal–ventral direction), with a mean value of 353.5 ± 58.21 µm compared to solid breast tumor cells (71 ± 5.25 µm; *P* < 0.0001, Fig. 2d). In addition, the total distance travelled was higher for leukemic (566.2 ± 81.53 µm; *P* < 0.0001) compared to breast cancer cells (232.1 ± 18.35 µm; *P* < 0.0001) (Fig. 2e). Next, we calculated the intravascular speed to be 193.6 ± 44.09 µm/s for the leukemic cells, and 63.6 ± 7.01 µm/s for breast cancer cells (*P* < 0.0001), indicating that the leukemic cells travelled significantly faster than breast tumor cells (Fig. 2f).

### Metastatic breast cancer cells can actively migrate and survive longer within the host

After injection, tumor cells generally migrated in the direction of blood flow as they were injected in the DoC. In order to discriminate whether tumor cells disseminated passively due to blood flow or migrated actively, we used silent heart morpholino^37^-injected zebrafish for xenotransplantation. In this no-blood-flow environment, we observed that even though most of the injected metastatic breast cancer cells remained at the site of administration, less than 10% of the cells migrated to the tail region (Figure S1), indicating that these cells are capable of active migration.

**Figure 3:**
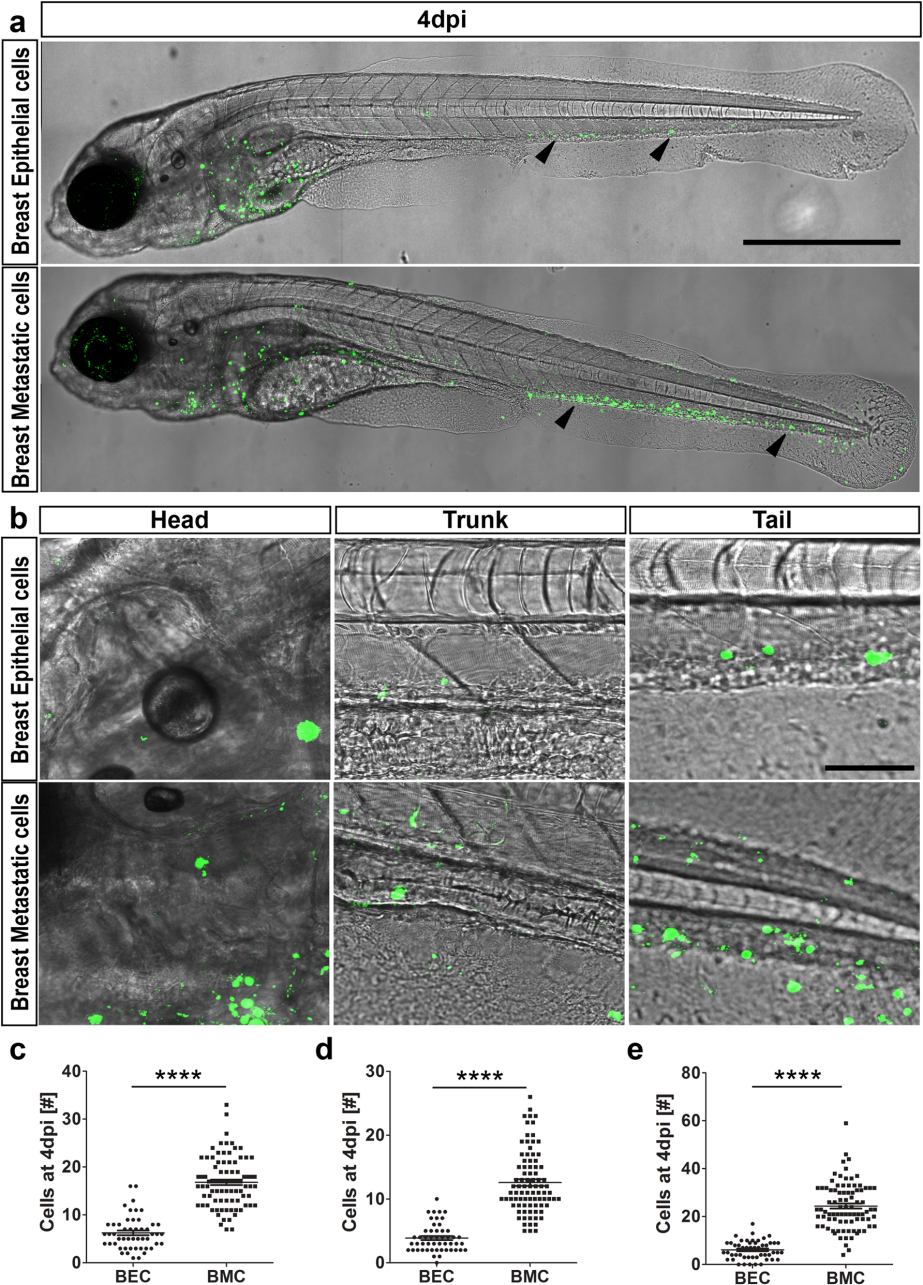
Dissemination of epithelial versus metastatic cells. (**a**) At 4 dpi, breast epithelial cell (BEC) numbers were drastically reduced compared to breast metastatic cells (BMC), as indicated by black arrowheads. Both breast epithelial and breast metastatic cells were depicted in green on a transmission image of zebrafish. Scale bar 500 µm. (**b**) Representative images of head, trunk, and tail regions of both BEC and BMC with high magnifications. Scale bar 50 µm. (**c**–**e**) Quantifications of the cells at 4 dpi in all the regions. Head (**c**), trunk (**d**), and tail (**e**) showed that breast metastatic cells survived better in the eZXM [N = 80 embryos each]. In all the regions observed, the cell numbers were significantly higher for breast metastatic cells. (**c**–**e**) Plots represent means ± sem. Statistical analyses: two-tailed Mann–Whitney’s *U*-test.

Next, to investigate whether the observed distribution is a specific feature of malignant cells, we xenotransplanted and traced malignant breast cancer cells (MDA-MB231) and non-malignant breast epithelial cells (MCF10A), in *casper* embryos. As reported previously^25^, much lower numbers of non-malignant breast epithelial cells survived compared to breast cancer cells over time (Fig. 3a), irrespective of whether these cells were located in the head, trunk, or tail regions (Fig. 3b). Quantification of cell numbers at 4 dpi revealed that the highly metastatic breast tumor cells persisted much longer in the host (53.78 ± 1.74%, *P* < 0.0001, for all regions), whereas the fluorescence signal of normal epithelial cells regressed after 48 h of transplantation (16.24 ± 0.99%), indicating engraftment failure of the non-malignant cells (Fig. 3c–e).

### Host immune cells react to injected cancer cells

**Figure 4:**
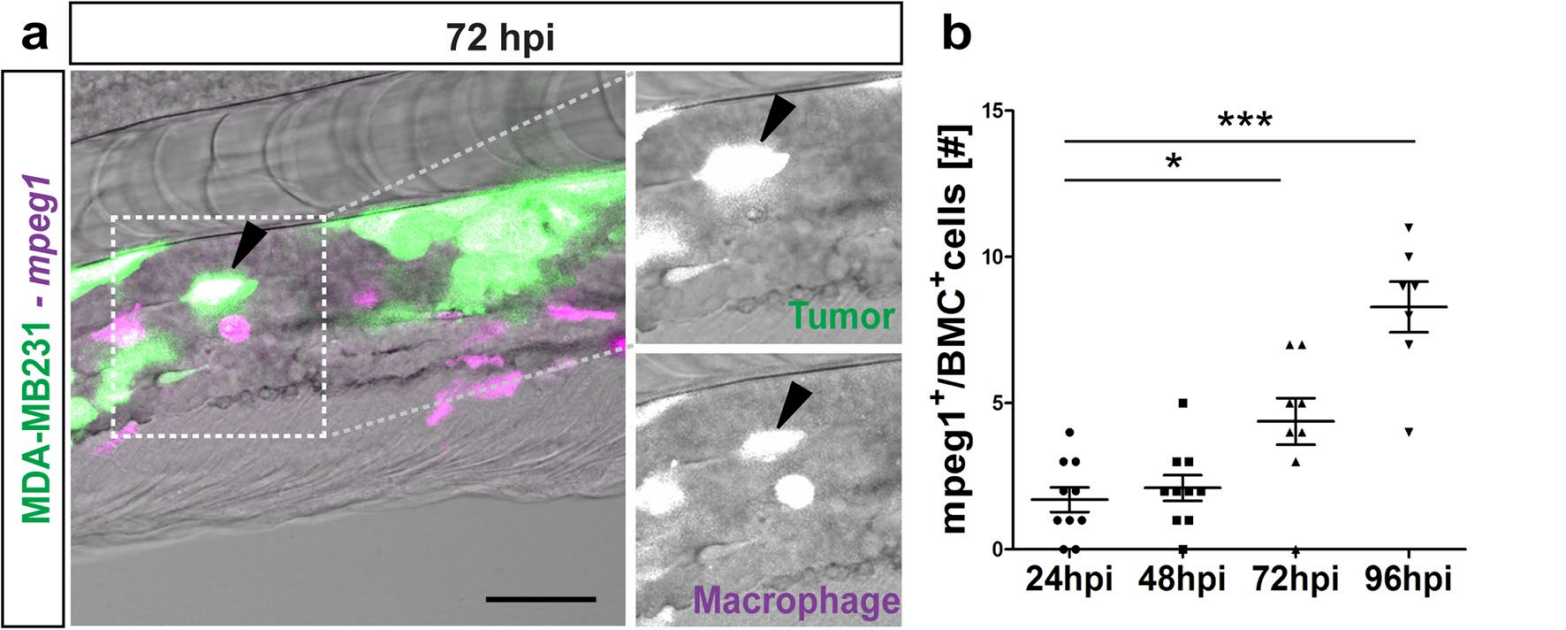
Macrophages react to tumor cells. (**a**) Representative image of GFP-labeled breast metastatic cells (BMC) (MDA-MB231) (in green) xenografted in eZXM expressing mCherry-labeled macrophages (in magenta). Co-localization of tumor cells with macrophages was observed (black arrowhead). Higher magnification of boxed region with MDA-MB231 cell (green—top) and macrophage (magenta—bottom). Scale bar 100 µm. (**b**) Quantification of the co-localization of tumor cells (BMC^+^) with macrophages (mpeg1^+^) over time. At 72 hpi, a significant increase in co-localization was observed. Plot represented means ± sem [N = 10 embryos were quantified for each time points: 24, 48 hpi and 96 hpi]. Statistical analyses: one-way ANOVA followed by Dunnett’s test for multiple comparisons. Multiple comparisons: 24 hpi vs. 48 hpi (*P* = 0.9225); 24 hpi vs. 72 hpi (*P* = 0.0103); 24 hpi vs. 96 hpi (*P* < 0.0001).

Cell numbers of injected metastatic breast cancer cells showed a tendency to decline from 1 dpi, with significant reduction to about 40–50% of the original cell number at 3 dpi, suggesting a reduced survival after xenotransplantation (Figure S2). Interestingly, high magnification SPIM time-lapse recordings revealed host-tumor interaction, wherein a host cell interacts with a tumor cell for  ~ 6 h finally enclosing tumor cell (Movie_3). Therefore, to test whether the host immune system is responsible for the observed reduction in injected tumor cells over time, we investigated the role of immune cells, specifically macrophages, by injecting metastatic MDA-MB231_eGFP (breast cancer) cells into the *Tg*(*mpeg1:mCherry*) zebrafish reporter line, in which macrophages are labeled by mCherry^38^. We observed co-localization of tumor cells with macrophages (Fig. 4a), and quantification of the number of double positive cells (macrophage co-localizing with a tumor cell) over time showed a significant increase in co-localized cells at 3 dpi (4.37 ± 0.80 cells) compared to 1 dpi (1.70 ± 0.42 cells,*P* = 0.0103; Fig. 4b). Additionally, macrophage ablation using *Tg*(*mpeg1:gal4-UAS:NTR-mcherry*)^38,39^ showed an improved tumor survival at 3 dpi (Figure S3a,b,c).

### Extravasation and caudal tail invasion by metastatic tumor cells

**Figure 5:**
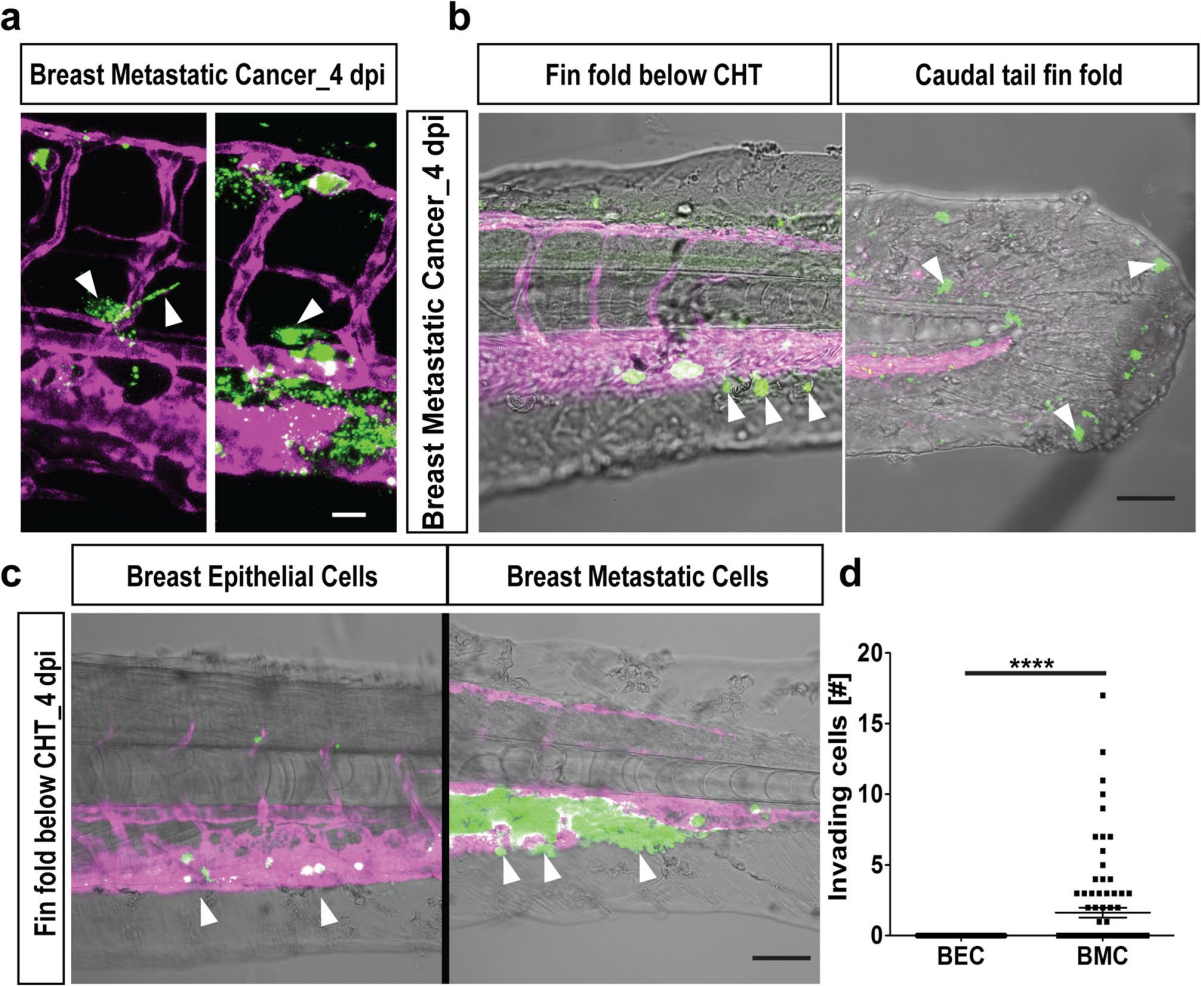
Extravasation and invasion of tumor cells. (**a**) Representative images of breast cancer cells (in green) initiating extravasation through the vasculature (in magenta) by forming protrusions (left, white arrowheads). These protruding cells later pushed the entire cellular content into the surrounding tissue (right, white arrowhead). Scale bar 20 µm. (**b**) Breast metastatic cells (in green) invaded the avascular tail region (vasculature in magenta). Two representative images of invading tumor cells (white arrowheads) in the fin-fold below the CHT region (left) and caudal tail fin-fold (right). Scale bars: 150 µm. (**c**) Representative images of invasion of cells out of the vasculature (in magenta) below CHT. Breast epithelial cells (in green) did not invade below CHT (white arrowheads, left panel) while few of the breast metastatic cells (in green) showed invasion below CHT (white arrowheads, right panel). Scale bar: 150 µm. (**d**) Quantification at 4 dpi revealed significant tail invasion of the breast metastatic cells (BMC) over breast epithelial cells (BEC) [N = 80 embryos each]. Plots represent means ± sem. Statistical analyses: two-tailed Mann–Whitney’s *U*-test.

We examined whether extravasation and tissue invasion by tumor cells, the hallmarks of tumor metastasis, could be observed in real-time using the eZXM. To visualize extravasation, we acquired high magnification time-lapse images of the tail region of the eZXM after they were injected with metastatic MDA-MB231 cells at the DoC. An extravasation event was recorded when tumor cells were found leaving the vessels and entering into the surrounding tissue, and high-resolution images provided a unique insight into the process of extravasation (Movie_4). Specifically, tumor cells appeared to change shape from ‘round’ to one with extending protrusions. After a few hours, these protrusions extended their filopodia-like structures into the tissue near the vessel wall. Additionally, confocal images of fixed embryos at 4 dpi revealed that these protruding cells near the vessel wall pushed their entire cellular contents outside the vessel wall, marking the completion of extravasation, as shown in Fig. 5a.

Next, we also assessed invasion behavior. The injected tumor cells were termed invading cells^25^ if they migrated outside the vasculature and into the avascular caudal tail region. At 4 dpi, metastatic MDA-MB231 cells appeared to invade the avascular fin-fold below the CHT and into the caudal fin fold (Fig. 5b). Importantly, metastatic breast tumor cells were capable of invading the avascular tail fin while normal breast epithelial cells did not, thus displaying characteristic tumorigenic behavior (Fig. 5c). Invasion at the caudal tail fin at 4 dpi was observed in 31.81% (28/88 injected embryos) of the MDA-MB231 injected embryos (Fig. 5d compared to 0% in MCF10A injected embryos (0/63 injected embryos; *P* < 0.0001). Additionally, injections of breast tumor cells having stable GFP expression showed similar dissemination, extravasation and invasion at the caudal tail fin as that of DiI labeled cells (Figure S4a,b). Further, primary tumor cells from breast cancer patients invaded the tail fin as early as 1 dpi in the eZXM (Figure S5).

### Rock-inhibition inhibits leukemic cell dissemination

**Figure 6:**
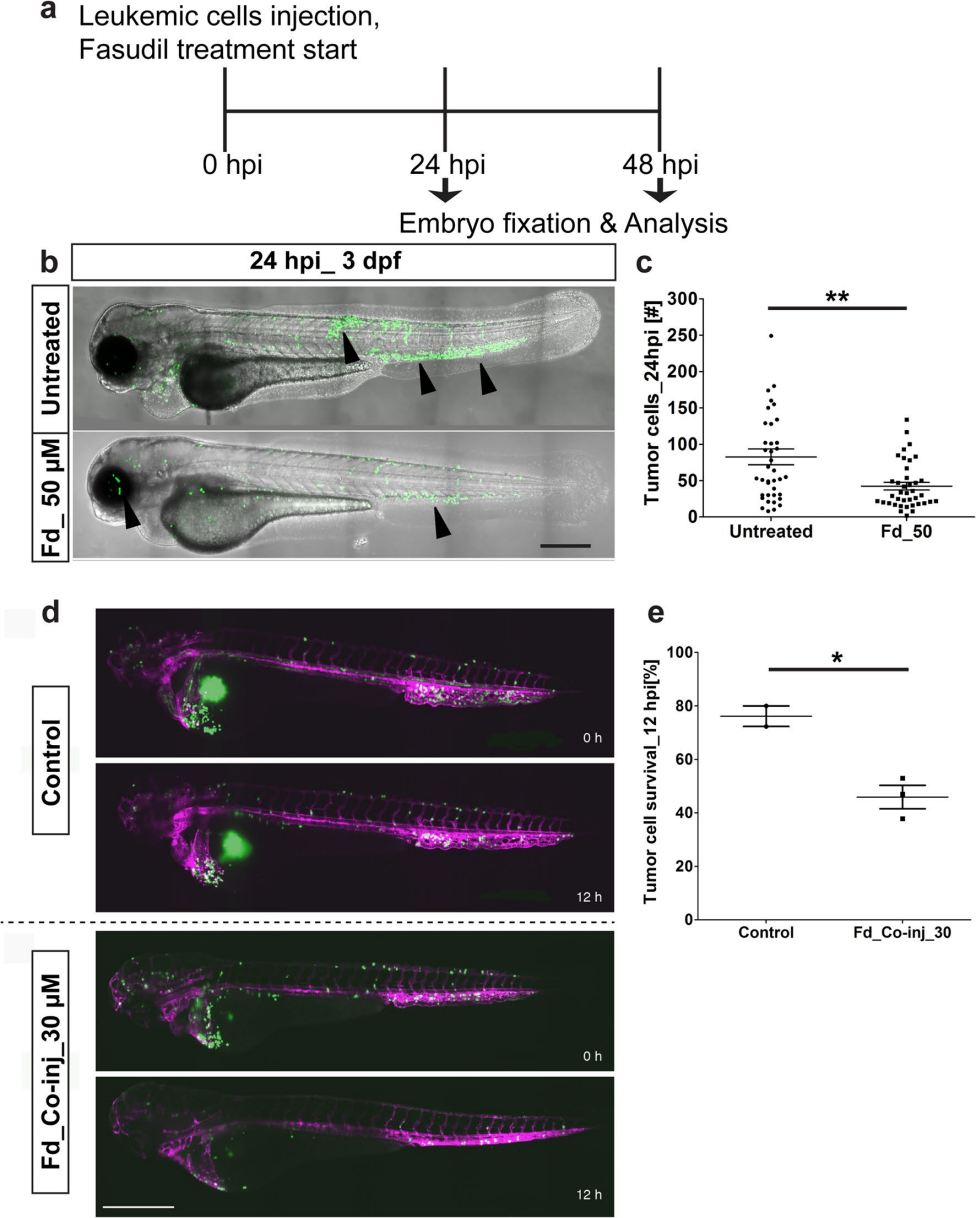
Effect of Fasudil on leukemic cells in the eZXM. Validation of the eZXM with the ROCK1 inhibitor Fasudil. (**a**) Schematic diagram depicting the experimental design: Leukemic cells (in green) were injected at 48 hpf, followed by the start of the Fasudil treatment to half cohort of the injected embryos and the other half were maintained in the normal E3 medium (control: untreated). At 24 hpi, few embryos were fixed and analyzed for tumor survival and at 48 hpi, remaining embryos were fixed and analyzed; (**b**) 50 µM Fasudil treatment (bottom) of the eZXM revealed a significant reduction (black arrowheads) in eGFP labeled leukemic cells (in green) at 24 hpi compared to untreated controls (top). (**c**) Quantification of tumor cells at 24 hpi showed a decrease in tumor cell number in 50 µM Fasudil-treated embryos [N = 40 embryos]. (**d**) Frames from the time-lapse movies showed dissemination of eGFP-tagged leukemic cells (in green) inside the eZXM expressing the vasculature marker *Tg*(*kdrl:Hsa.HRAS-mCherry*) (magenta) at the beginning of the experiment (0 h) and after 12 h (12 h) in treated and untreated fish. (**e**) Quantified survival rate of the leukemic cells as observed from the in vivo time-lapse movies (right). Scale bar 500 µm. (**c**, **e**) Plots represent means ± sem. Statistical analyses: (**c**) two-tailed Mann–Whitney’s *U*-test, (**e**) Welch Two Sample t-test.

To evaluate the utility of the eZXM to monitor the effect of a compound which interferes with cell motility, we studied the effects of the ROCK1 (Rho-associated coiled-coil protein kinase 1) inhibitor Fasudil in our model using leukemic tumor cells. As our colleagues have demonstrated that ROCK1 inhibition suppresses leukemic growth in vitro and engraftment in a long-term murine xenotransplantation model^40^, we set out to study its effects in our in vivo set-up. As a first step, we found that Fasudil treatment up to a concentration of 100 µM did not lead to phenotypic defects in treated embryos, compared to untreated embryos (Figure S6a). Additionally, in vitro Fasudil treatment (50 µM) of leukemic (OCI-AML3) cells showed a reduction of 69% in their metabolic activity after 24 h of treatment (Figure S6b) and PI/Annexin staining with flow cytometry analysis revealed that around ~ 50% of leukemic cells were apoptotic at a Fasudil concentration of 50 µM (24 hpt; Figure S6c). To study the effect of Fasudil in our eZXM, tumor cells were injected at 2 dpf *casper* embryos and at 2 hpi half of the injected embryos were treated with Fasudil as shown in the scheme (Fig. 6a). In vivo, in the leukemic tumor cells injected embryos, we found that, compared to the untreated control embryos at 24 hpi (82.67 ± 11.02), Fasudil-treated embryos showed a significant reduction in tumor cell numbers (42.36 ± 5.14, *P* = 0.0019; Fig. 6b, c). Additionally, this reduction in the Fasudil-treated embryos (33.30 ± 4.86, P = 0.0179; Figure S6d) was found to be significant at 48 hpi. Moreover, to validate SPIM as a screening platform, we performed a pilot experiment wherein leukemic tumor cells were co-injected with 30 µM Fasudil in eZXM embryos. Both the control and Fasudil co-injected embryos were imaged simultaneously. Visual comparison of the control and Fasudil-treated embryos suggested that Fasudil indeed decreased leukemic tumor cell survival in zebrafish (Fig. 6d). Quantification of all cells inside the xenografted embryos (cells inside the yolk were excluded) at the start of the experiment (0 h) and after 12 h showed that 76.15% ± 3.85% (mean ± SEM, n = 2) of all cells survived in controls, while only 45.92% ± 4.40% (mean ± SEM, n = 3; *P* = 0.0355) survived in Fasudil co-injected embryos (Fig. 6e). These observations imply that the eZXM model described here could be used as functional screening platform to identify compounds interfering with tumor cell spread and invasion.

## Discussion

Over the past decades, different techniques have been developed to understand the mechanisms underlying the process of metastasis as it is the major determinant of cancer-related morbidity and death. Despite the golden standard being the choice of using immune-permissive mouse models for cancer research and potential drug screening, there are several inherent disadvantages associated with murine xenotransplantation models^41,42^. These limitations include long duration for tumor cell engraftment and growth, in vivo variation in growth, expensive housing and the difficulties associated with live imaging for visualizing engraftment. Specifically, continuous visualization of cells to track tumor spread or metastasis is not possible. The eZXM on the other hand, can overcome most of the limitations and is a useful model to investigate metastasis as reported by others previously^19,20,25,28,43,44^. Here, we describe how SPIM, a rapid, efficient, and non-invasive imaging approach^45^, can be used to visualize and understand the metastatic cascade in vivo. We chose SPIM as it captures the spatiotemporal dynamics of individual tumor cells in circulation with high precision and resolution, and used it to continuously track the in vivo behavior of two different metastatic cell lines in the eZXM. Despite tumor cells spreading throughout the embryo due to passive dissemination via blood flow, MDA-MB231 cancer cells also migrated actively in the absence of blood flow (silent heart morpholino), and SPIM time-lapse images revealed acute differences in migration patterns among all tested cell types. The observed migration of the fluorescent solid cancer cells through circulation to distant sites in the embryo was similar to that reported previously^26^. However, after 5 hpi, breast cancer cells were predominantly adherent in the tail, especially in the CHT region. This finding might be explained by abundant CXCL-12 (a chemokine) expression in the CHT and its association with the homing of metastatic breast tumor cells^26^. In contrast, leukemic cells mostly remained in circulation, compliant with their origins, and their migration in the eZXM can best be described as ‘flying’ due to the swift intravascular migration. However, the characteristic differences observed in in vivo migration were specific for the cancer cell subtype mentioned, and further experiments with other subtypes are required to generalize these observations to establish a characteristic profile of breast and leukemic tumors. Additionally, cardiac edema was observed as a consequence of injections in 30–40% of the injected embryos starting from 3 dpi with diverse degree of severities (minor to significant ones).

High-resolution SPIM data also facilitated the analysis of tumor cell morphology. Solid tumor cells are generally adherent in nature and adapt to different environmental conditions by switching their morphology and migratory mode^46^. Interestingly and concurrent with the above observation, solid tumor cells demonstrated a change in cell shape from round to amoeboid with a protruding arm, which helped their migration from the dorsal longitudinal anastomotic vessel to the intersegmental vessels. This phenomenon also explains the nature of tumor cells to adapt to their environment to stay motile^47^. Distinctly, the leukemic cells failed to show this amoeboid and protruding nature, as expected from their relatively smaller size and non-adherent nature. Further, when a large number of cancer cells (~ 500 cells) were injected into the DoC, time-lapse images revealed huge protrusions and network like formations between neighboring cells, probably to establish stable homotypic contacts that are crucial to promote collective migration and tumor progression (unpublished data/data not shown).

Quantitative analysis of tumor dissemination in the eZXM after transplantation and related aspects including total number of tumor foci, tumor cell burden, average and cumulative distance traveled from the injection site have been previously characterized^43,48^. Many investigators have also used intravital microscopy to measure these parameters in vivo in mouse tissue after invasion^46^. Nevertheless, continuous in vivo monitoring of the cells and associated changes in quantitative parameters has not been reported, and a semi-automated, in-house developed tracking platform used to quantify SPIM observations revealed that total, net and maximum distance travelled were always higher for leukemic cells compared to breast cancer cells. Also, we have calculated for the first time, the intravascular speed in anterior–posterior and dorsal–ventral direction of tumor cells in the eZXM, and as expected, leukemic cells were much faster than the barely-migrating breast adenocarcinoma cells. Most likely due to the larger cell diameter, breast cancer cells get stuck in smaller vessels of the zebrafish embryos, thereby affecting their intravascular speed. However, analysis of instant speed of these cells at different sites showed there is no specific association of speed with where the tumor cells are in fish (data not shown) and cells migrate by squeezing through the vessels. Although some of the parameters included in our study have been chosen arbitrarily, we believe that distance and speed may be seen as a surrogate for intra-versus extravascular distribution, cell size and adhesiveness as well as migratory activity. These quantitative parameters will also help in identifying drugs that might interfere in migration and invasion by evaluating differences in the distance, speed values between the control and drug treated embryos.

Despite the immune-tolerant state of embryonic tissue, metastatic breast tumor cell numbers started to decline around 72 hpi. Tumor infiltration by macrophages during metastasis is a known phenomenon^1^, and a previous study has demonstrated that host macrophages can recognize and kill xenogenic tumor cells^49^. In concordance, high-resolution SPIM images revealed engulfment of a breast cancer cell by a perivascular host cell and increased co-localization of these tumor cells and host macrophages at 3 dpi, suggesting that this might be the response of host macrophages to the engrafted tumor cell. We speculate that there is a direct link between the increase in co-localized cells and the reduction in tumor cell number at 4 dpi. Functional inhibition of macrophages using MTZ-NTR system in the *Tg*(*mpeg1:gal4-UAS:NTR-mcherry*)^38,39^ supported our speculation by revealing improved tumor survival, thereby confirming a potential role for innate host immune cells in restricting the survival of injected human tumor cells. However, the mechanism behind the reduction in cell numbers at 4 dpi and the clearance of tumor cells remains elusive. Further experiments are required to identify the exact role of host immune cells in the clearance of the engrafted tumor cells.

Previous reports have investigated the invasion and micrometastatic properties of xenografted cells in the caudal tail fin^28^, the dynamics of tumor cell extravasation, and associated tumor cell-endothelial cell interactions to remodel the vasculature^50^. We speculate that the cells with protrusions near the vessel wall mark the initiation of extravasation followed by complete extravasation of the cell into the tissue. Invasion at the caudal tail fin fold and fin folds below CHT by the metastatic breast tumor cells, but not the breast epithelial cells, attests to their tumorigenic property. Most importantly, the observed bio distribution of malignant cells could be partly confirmed using primary patient-derived tumor cells, thus supporting the relevance and potential clinical applicability of the described model. The zebrafish embryo could, therefore, complement existing patient-derived murine xenograft models.

Finally, we have applied Fasudil^40^, a ROCK1 inhibitor which is not used clinically but has been shown to have antileukemic effects in an RNA-interference screen. In line with this report, we observed a 42% reduction in leukemic cells in the Fasudil-treated embryos at 48 hpi. These findings suggest that our model might be especially suited to monitor the effect of compounds which specifically target tumor cell motility. Importantly, the intravascular distance and speed measurements reported here based on the SPIM movies might provide novel insight into the mode of action of different compounds. In contrast with the current treatment strategies involving non-specific cytotoxic drugs, we envision that our quantitative screening strategy will help screen for drugs that may interfere with adhesion and migration of metastatic tumors.

To conclude, we show that tumor cells retain their defining characteristics even after injection into the eZXM, making the eZXM a useful screening tool. Further, tumor cell dissemination characteristics described here can be used to gain insight into mechanisms of anti-tumor action of drugs. Therefore, we propose a combinatorial approach of using the eZXM with in vivo SPIM imaging as a functional screening platform that can complement current drug screening and personalized anti-tumor strategies.

## Methods

### Animal care and handling

The zebrafish (*Danio rerio*) strains were kept under standard conditions (28 °C in E3 buffer) until 48 hpf as described previously^51^. All experiments were performed in accordance with the local and national regulations and guidelines and were approved by the local institutional review board (Ethical committee of Technische Universität Dresden, Fiedlerstraße 33, 01307 Dresden). All zebrafish experiments and procedures were performed in accordance with the Guidelines for Care and Use of Laboratory Animals of *the Federal Republic of Germany *(*Tierschutzgesetz*), approved by the Landesdirektion Sachsen (DD24-5131/346/11, DD24-5131/346/12 and TVV 8/2020), by the institutional animal ethics committee of the Technische Universität Dresden, and in accordance with *EU Directive 2010*/*63*/*EU.*

### Cells and cell culture

Breast cancer cells (MDA-MB231) and non-malignant breast epithelial cells (MCF10A) were obtained from the German Collection of Microorganisms and Cell Cultures (DSMZ, Braunschweig, Germany) and from ATCC (LGC Standards GmbH, Wesel, Germany), respectively. All the cell lines were obtained between 2011 and 2013, and authenticated by DNA profiling using 8 different and highly polymorphic short tandem repeat (STR) loci and were cultured as described previously^52^.

We established and standardized a protocol for the isolation of primary tumor cells from breast cancer patients (n = 20, tumor stages 1 and 2) using a tumor dissociation kit (130-095-929) and a tumor cell isolation kit (130-108-339) (both from Miltenyi Biotec). The isolated cells were characterized by immunofluorescence staining for pan-cytokeratin and immunophenotyping for CD24/CD44.

The use of primary tumor cells from patients after informed consent had been approved by the institutional Review board of the University Hospital Dresden (Ethical approval no. EK 32012016, EK221102004, EK47022007; Ethical committee of University Hospital, Technische Universität Dresden, Fiedlerstraße 33, 01307 Dresden).

To obtain the human leukemic cell line OCI-AML3_eGFP, we first produced lentiviral vector particles by transfecting HEK293T cells with the lentiviral vector pRRL.SIN.cPPT.SFFV.GFP.WPRE^53^ along with the packaging plasmids psPAX and pVSVg using polyethylenimine (PEI). Lentivirus-vector containing media was collected 48 h after transfection. OCI-AML3 cells were then infected with lentiviral vector particles (0.5× viral supernatant) in the presence of 1 mg/mL protamine, GFP expression was evaluated by flow cytometry, and positive clones were sorted using the BD FACSAriaTM II cell sorter (BD Biosciences).

### Human tumor cell preparation for transplantation and microinjection

Tumor cells were labeled with the fluorescent cell tracker CM-DiI (a lipophilic tracer, Invitrogen). A cell suspension was prepared from a 70–80% confluent monolayer as follows. Cells were trypsinized using trypsin–EDTA (0.05%), washed once in complete medium, centrifuged at 1,200 rpm for 8 min, re-suspended in PBS, and 4 µl of CM-DiI added. The cell suspension was incubated in a 37 °C water bath for 4 min, immediately transferred to ice for 20 min, centrifuged for 5 min at 1,200 rpm, and the cell pellet suspended in transplantation buffer at 100–150 cells/1 nl. *Casper* embryos (45hpf) were manually dechorionated and anesthetized using 0.02% tricaine and transferred to a petri dish containing 1.5% low melting agarose in E3. Tumor cells tagged with CM-DiI were loaded in a glass capillary and micro-injected into the blood circulation of multiple zebrafish lines (*casper*^15^, [*Tg*(*kdrl:EGFP*)^*s843*^]^33^ and *Tg*(*kdrl:Hsa.HRAS-mCherry*)^34^ via the duct of Cuvier (DoC). Engrafted embryos were maintained in a new petri dish at 33 °C. Based on the fluorescence spread of the injected embryos at 2 h post injection (hpi), embryos with tumor cells in the blood circulation were selected for experiments.

### Image acquisition and processing

In order to analyze migration of injected cancer cells, live imaging of the engrafted embryos was carried out using a multi-sample, multidirectional (mSPIM)^35,36^ selective plane light sheet illumination microscope. Injected embryos were imaged with SPIM for approximately 30 h at 7× magnification with images acquired every 10 min. For high-resolution imaging of extravasation, only the tail portion of the embryo was imaged with 14× magnification. To ensure constant temperature, a perfusion chamber, maintaining 33 °C, was installed. Images were later stitched and processed using in-house developed Image J plugins^36^, that are freely available at: https://github.com/DaetwylerStephan/multi_sample_SPIM. Sample drift was corrected with a rigid registration using the image registration software elastix^54,55^. For quantification, embryos were fixed in 4% paraformaldehyde at 4 °C overnight. Fixed embryos were imaged using inverted confocal microscopy (Zeiss LSM 780) at 20× magnification (whole embryos) or at 40× magnification (tail region). Confocal stacks were converted to maximum intensity projections using Image J (v 1.51h). The quantification of engraftment of tumor cells or tumor cell survival was done manually with confocal microscopy. At 4 dpi, tumor cell numbers in the head, trunk and tail region were counted. Manual quantification allowed us to inspect and resolve clustered cells by going through each and individual z plane. For obtaining the percentage of cells with active migration, the initial number of tumor cells injected was measured and compared to manually quantified number of cells migrated in the tail.

### Tracking analysis

An in-house developed tracking method was used to analyze the maximum intensity projections of the time-lapse images generated by the mSPIM. This semi-automated tracking analysis combines three already existing and broadly-used open-source software tools, namely CellProfiler (v. 2.1.1)^56^, CellTracker^57^ and R (v. 3.1.2,CRAN,R Core Team, 2014). CellProfiler was used for image segmentation as well as for an automated pre-tracking step. The resulting data was thereafter transcribed into a .xml file using R. Potential mistakes that occurred during the automated tracking process could then be corrected with the help of CellTracker, before the data was finally analyzed and visualized using R. Several measurements could be extracted from the resulting migration data. In the presented work, we concentrate on the maximum distance between any two points in the migration path, the net distance between the final position and the injection site (origin), the total distance travelled as well as the migration speed of the cells.

### Macrophage ablation and drug treatments

For efficient macrophage ablation experiments, the double transgenic larvae *Tg*(*mpeg1:Gal4*^38^, *UAS:NTR-mCherry*^39^, were screened for strong mCherry expression at 24 h post fertilization (hpf) and incubated in E3 medium supplemented with metronidazole (MTZ, Sigma-Aldrich). Fresh MTZ was prepared using 0.2% DMSO in E3, at 5 mM and 10 mM concentration prior to incubation. mCherry positive embryos at 24 hpf were separated into 3 groups and then incubated as follows: (1) Control, (0.2% DMSO); (2) 5 mM MTZ and (3) 10 mM MTZ treatment. Embryos were incubated in the dark as MTZ is light sensitive. At 48 hpf, all three groups were injected with stable GFP transfected breast tumor cells (MDA-MB231-EGFP). MTZ treatment of 24 h prior to injection was then followed by an addition of 4 days (until 4 dpi). Medium was changed for all the groups every day and fresh MTZ was provided. Ablation efficiency and tumor survival was analyzed using confocal fluorescent microscopy.

### Drug treatment and efficiency evaluation

Functional validation of the model was carried out using the Rock-inhibitor, Fasudil^40^. A working concentration of 50 µM Fasudil in water, prepared from 1 mM stock, also in water, was added to E3 medium containing injected embryos. Embryos were maintained for 2 dpi in the E3 medium containing Fasudil. Tumor cell survival at 24 and 48 h post injection (hpi) was assessed by manual counting of the fluorescent tumor cells with confocal microscopy. Tumor cell quantification from SPIM time-lapse at 12 hpi was done using ImageJ and was normalized to 0 hpi baseline.

### Phenotypic assessment of drug-treated embryos

48 hpf *casper* embryos were separated into 4 groups (n = 20 embryos/group) namely control (untreated), 10 µM treated, 50 µM treated, and 100 µM Fasudil treated embryos. Fasudil was added externally to the medium and the embryos were incubated at 33 ℃ for the duration of the experiment. The embryos were assessed each day for phenotypic toxicity until 4 days post treatment (4 dpt) and imaged live in an Olympus MVX10 microscope.

### MTT assay

Fasudil treated and untreated tumor cells (leukemic cells) were analyzed for their metabolic activity using MTT Cell Proliferation Assay Kit (provided by ATCC® 30-1010 K). The yellow tetrazolium MTT (3-(4, 5-dimethylthiazolyl-2)-2, 5-diphenyltetrazolium bromide) is reduced by metabolically active cells, to intracellular purple formazan that can be solubilized and spectrophotometrically quantified at 570 nm. Cells were incubated with the drug for 24 and 48 hpt. The MTT reagent was added to the treated and untreated cells and manufacturer’s recommendations were followed^58^. Absorbance was plotted and the metabolic activity of the cells was determined.

### Annexin/PI staining of Fasudil treated leukemic cells

Apoptosis in Fasudil treated cells, along with their respective untreated leukemic controls, was analyzed using the APC Annexin V Apoptosis Detection Kit with PI (BioLegend). Manufacturer’s instructions were modified as follows. Cells were washed twice with cold BioLegend’s cell Staining Buffer (centrifuged at 1,200 rpm, 8 min 4 °C) and then resuspended in Annexin V Binding Buffer at a concentration of 0.5–1 million cells/ml. All other steps in the protocol were followed as per instructions along with recommended concentrations of PI and APC Annexin V. Cells were analyzed by flow cytometry with appropriate gatings.

#### Statistical analysis

Statistical analysis was performed using the Prism software (Ver.6.0, GraphPad La Jolla, USA). Results are expressed as the mean  + /− SEM. If not indicated otherwise, student’s t-test or one-way analysis of variance (ANOVA) were performed followed by the Dunnett’s method for multiple comparisons. For all tracking measurements, Kruskal–Wallis one-way analysis of variance were performed followed by post hoc Dunn’s method for multiple comparisons. *P* < 0.05 was considered to be statistically significant (*0.01 < *P* < 0.05; **0.001 < *P* < 0.01; ***0.0001 < *P* < 0.001; ****P < 0.0001).

## Supplementary information

Supplementary Video 1

Supplementary Video 2

Supplementary Video 3

Supplementary Video 4

Supplementary information
