# Supplementary material for: Long-term in vivo imaging reveals tumor-specific dissemination and captures host tumor interaction in zebrafish xenografts
Source: Sci Rep. 2020 Aug 6;10:13254. doi: 10.1038/s41598-020-69956-2 (PMC7411039; doi:10.1038/s41598-020-69956-2)
Supplement: Supplementary file 5 — Supplementary information [file 41598_2020_69956_MOESM5_ESM.pdf]

Supplementary Information

Long-term *in vivo* imaging reveals tumor-specific dissemination and captures host tumor interaction in zebrafish xenografts

Nandini Asokan[1,2], Stephan Daetwyler[3], Stefanie N Bernas[1,4], Christopher Schmied[4], Steffen Vogler[4], Katrin Lambert[1,2], Manja Wobus[2], Martin Wermke[2], Gerd Kempermann[1,4], Jan Huisken[3,#a], Michael Brand[1*], Martin Bornhäuser[1,2,5,6*]

[1]Center for Regenerative Therapies Dresden (CRTD), Technische Universität Dresden, Germany;

[2]Division of Hematology, Oncology and Stem Cell Transplantation, Department of Medicine I, University Hospital Carl Gustav Carus, Technische Universität Dresden, Germany;

[3]Max Planck Institute of Molecular Cell Biology and Genetics (MPI-CBG), Dresden, Germany;

[4]German Center for Neurodegenerative Diseases (DZNE) Dresden, Germany;

[5]National Center for Tumor Diseases (NCT), Dresden, Germany;

[6]German Consortium for Translational Cancer Research (DKTK), DKFZ, Heidelberg, Germany;

[#a]Current Address: Morgridge Institute for Research, Madison, USA

*Corresponding Authors

Supplementary figures and legends

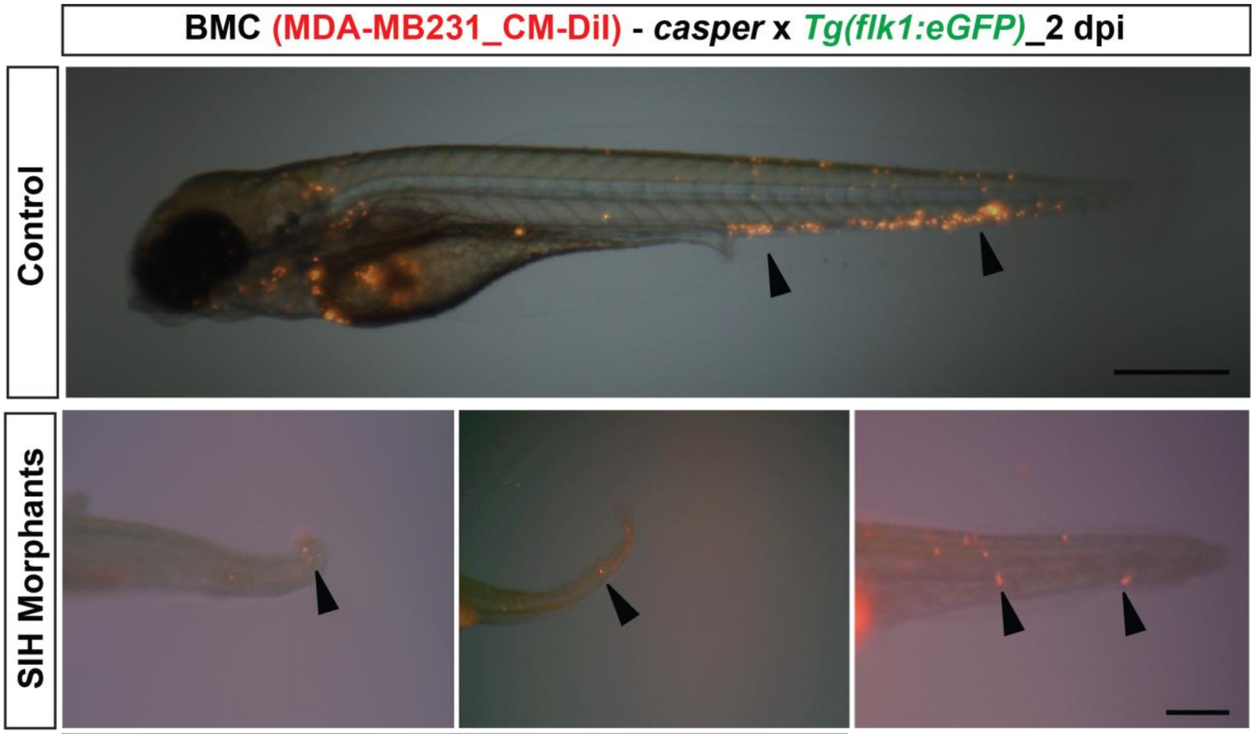

Figure S1. Active migration of the tumor cells. Breast metastatic tumor cells (BMC) labeled with CM-DiI were injected into silent heart morpholino injected eZXM. Silent heart morpholino stopped the heartbeat. Representative image of control morpholino eZXM injected with breast tumor cells (black arrowheads) with dissemination throughout the embryo (top). In the no-flow environment, breast tumor cells (black arrowheads) migrated actively from the site of administration towards the tail region (bottom).

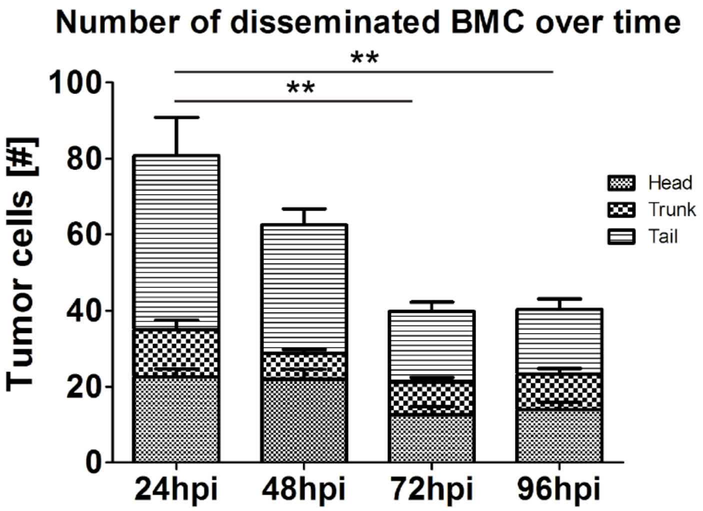

Figure S2. Dissemination of metastatic breast tumor cells and tail invasion of primary breast tumor cells. Breast metastatic tumor cells (BMC- MDA-MB231) were injected into the eZXM and the number of surviving cells were quantified over time. Quantification revealed that at 72 hpi, breast tumor cell numbers were significantly reduced compared to at 24 hpi. Plot represented means ± sem [N=10 embryos each]. Statistical analyses: one-way ANOVA followed by Dunnett's test for multiple comparisons. Multiple comparisons: 24hpi vs. 48hpi ($P > 0.9999$); 24hpi vs. 72hpi ($P = 0.0028$); 24hpi vs. 96hpi ($P = 0.0086$).

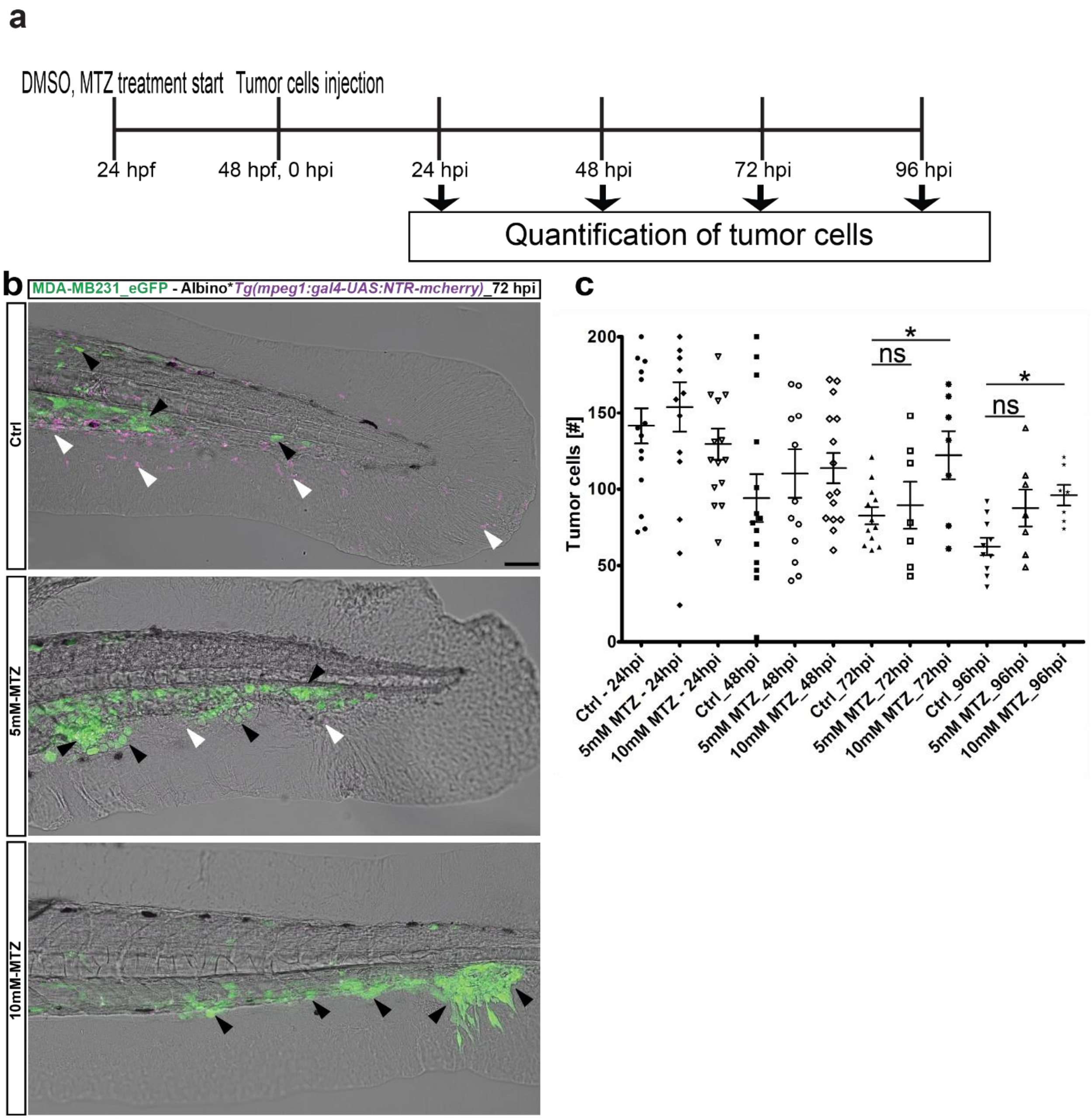

Figure S3. Ablation of macrophages revealed improved tumor cell survival. (a) Scheme depicting the experimental design: mCherry positive embryos were treated with either DMSO (for control) or MTZ (concentration: 5mM and 10mM) from 24 hpf. Every day, the embryo medium was changed with fresh DMSO and MTZ solution. 10 mM MTZ treatment resulted in 90% ablation of macrophages. Tumor cells were injected at 2 dpf and followed until 96 hpi. Every day embryos were analysed for tumor cell survival using confocal microscopy. (b) Representative image of GFP-labeled breast metastatic cells (BMC) (MDA-MB231) xenografted in eZXM expressing

mCherry-labeled (magenta) macrophages at 72 hpi. In the top, control panel (ctrl), macrophages (magenta - white arrowhead) was observed in great numbers along with tumor cells (green – black arrowhead). 5mM-MTZ treatment showed hardly 2-3 macrophages (magenta - white arrowhead) were found and tumor cell number were comparatively higher to top control panel. 10mM-MTZ treatment in the bottom panel showed improved tumor cell numbers and almost no macrophages. Scale bar 100 µm. (b) Quantification of the tumor cells (BMC^+) over time. At 72 hpi, a significant increase in the tumor cell survival was observed in the 10mM MTZ treated group compared to the control DMSO group. Plot represented means ± sem. Statistical analyses: one-way ANOVA followed by Dunnett's test for multiple comparisons. Multiple comparisons: Ctrl_72hpi vs. 5mM MTZ_72hpi (P = 0.8699); Ctrl_72hpi vs. 10mM MTZ_72hpi (P = 0.0335); Ctrl_96hpi vs. 5mM MTZ_96hpi (P = 0.0667); Ctrl_72hpi vs. 10mM MTZ_72hpi (P = 0.0136).

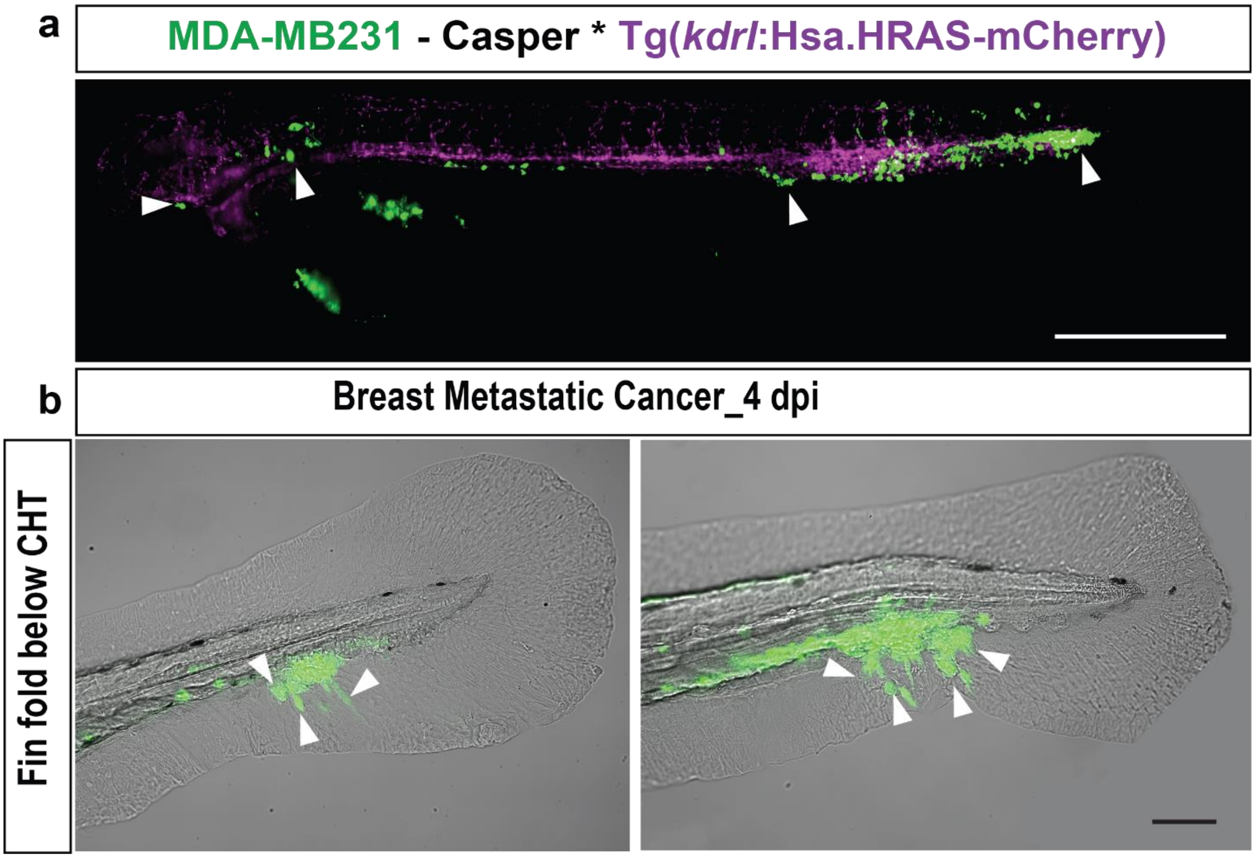

Figure S4. Dissemination and tail invasion of metastatic breast tumor cells with stable GFP expression. (a) Representative image of eZXM expressing the vascular marker *Tg(kdrl:Hsa.HRAS-mCherry)* in the *casper* background injected with eGFP labeled breast tumor cells (MDA-MB231_eGFP). The cells disseminated throughout the embryo as indicated by the white arrowhead. Vasculature in magenta, breast tumor cells in green; scale bar: 500 µm. (b) Representative images of breast tumor cells initiating extravasation by forming protrusions (left, white arrowheads). Breast metastatic cells invaded the avascular tail region. Representative images of invading tumor cells (white arrowheads) in the fin-fold below the CHT region. Scale bars: 100 µm.

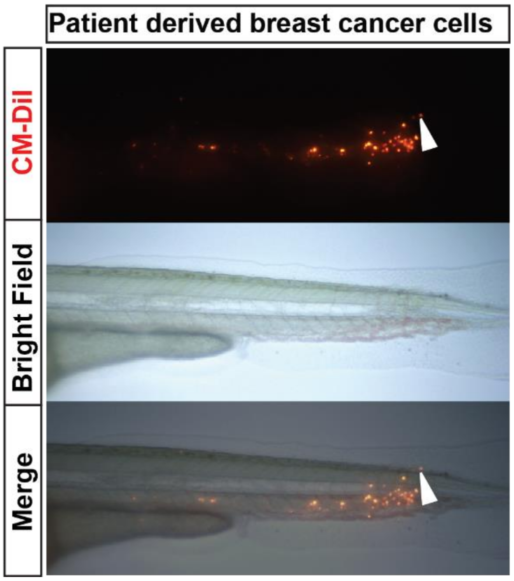

Figure S5. Tail invasion of primary breast tumor cells. Primary tumor cells (top) were labeled with CM-DiI and injected in the eZXM. Imaging of the tail part of at 1 dpi (bright field image, middle), revealed invasion of the tail fin fold region by a single cell (white arrowhead).

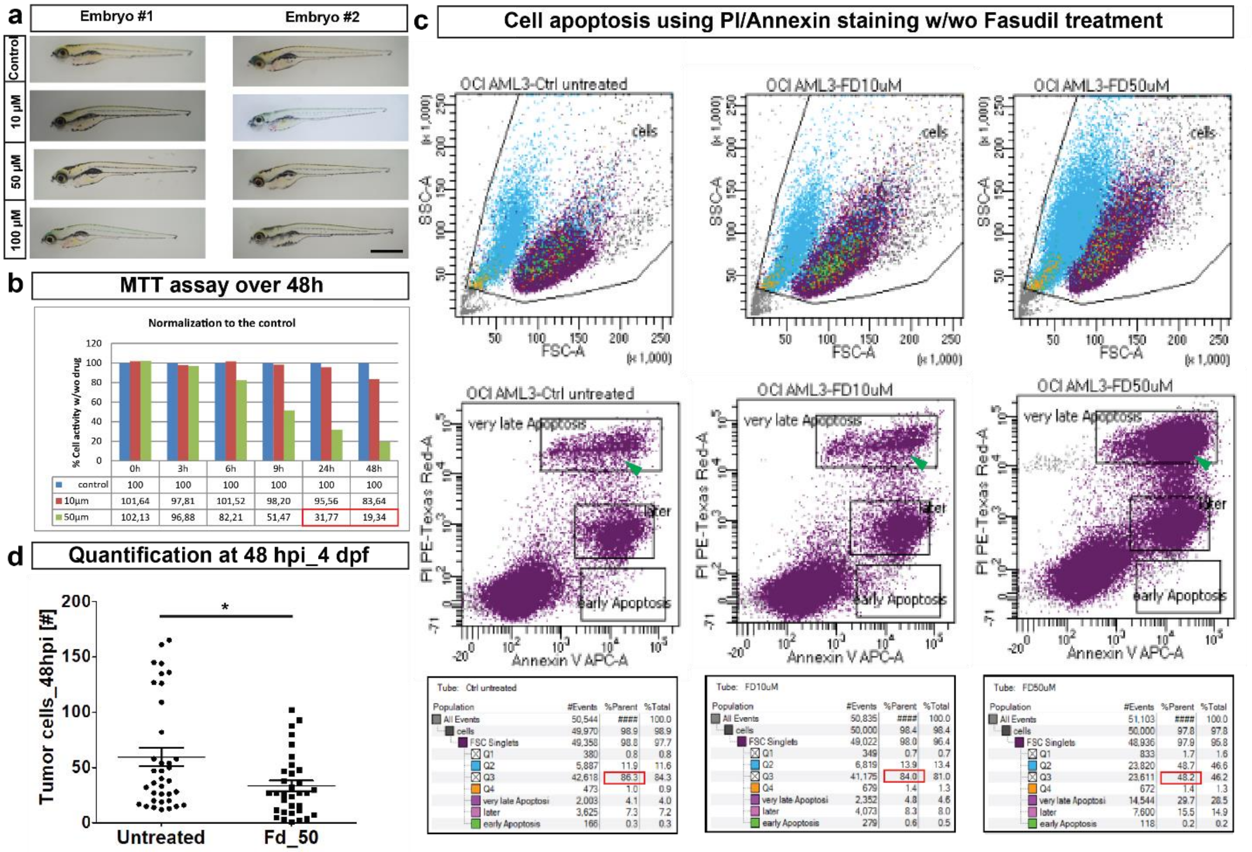

Figure S6: *In vitro* and *in vivo* effect of Fasudil. (a) Representative image of two embryo with different concentrations of Fasudil treatment to assess the phenotypic effect. Until 3 days post treatment (dpt), embryos did not show any phenotypic abnormality due to Fasudil treatments in comparison to control untreated embryos. Scale bar: 50 µm. dpt - days post treatment. (b) In *in vitro* culture, leukemic cells treated with Fasudil for 48 h showed a reduction of 80 % in their metabolic activity at 50 µM concentration using MTT assay (indicated by the values in red box). (Error bars are not indicated as experiment was performed only twice, a representative plot is shown here). (c) PI/Annexin staining on leukemic cells treated with Fasudil in *in vitro* for 24 h showed cells were apoptotic in comparison to control (left) as indicated by the green arrowhead in the second row of panel. Around 50 % of cells were only remaining after 50 µM Fasudil treatment as indicated by the red box in third row (right most bottom panel). (d) Quantification of tumor cells at 48 hpi *in vivo* in the zebrafish embryo showed a decrease in tumor cell number in 50 µM Fasudil-treated embryos [N=36 embryos]. Plots represent means ± sem. Statistical analyses: two-tailed Mann-Whitney's *U*-test

Supplementary movie legends

Movie_1. Dissemination of leukemic cells. OCI-AML3_eGFP cells (leukemic cells; magenta) were injected into the eZXM expressing the vasculature marker *Tg(kdrl:Hsa.HRAS-mCherry)* (depicted with green). Leukemic cells were migrating back and forth and mostly preferred to stay in circulation. Time shown as h:min:sec. Scale bar: 500 µm.

Movie_2. Dissemination of breast metastatic cancer cells. MDA-MB231 (breast cancer cells) labeled with CM-DiI (magenta) were injected into eZXM expressing the vasculature (green) marker *Tg(kdrl:EGFP)*^{s843}. After injection, the tumor cells migrated along with the blood flow, disseminated from head to tail, migrated towards the circulatory loop end, and adhered near the caudal hematopoietic tissue (CHT) region. Time shown as h:min:sec. Scale bar: 500 µm.

Movie_3. Host-cell enclosing a tumor cell. Metastatic breast tumor cells (green) were injected in eZXM expressing the vasculature marker *Tg(kdrl:Hsa.HRAS-mCherry)* (magenta). A tumor cell interacted with a host cell (magenta, white arrowhead). The host cell enclosed the tumor cell at 6 h after establishing contact. Time shown as h:min:sec. Scale bar: 50 µm.

Movie_4. 3D rendering of extravasation initiation. Breast metastatic cells (MDA-MB231_eGFP, in green) were injected in eZXM expressing the vasculature marker *Tg(kdrl:Hsa.HRAS-mCherry)* (magenta). 3D rendering of time-lapse SPIM movie showed a cell having a tendency to extravasate by forming protrusions (white arrowhead). Protrusion projected their arms into the surrounding lumen marking the initiation of extravasation event.
